# Gender Differences in Patients With COVID-19: Focus on Severity and Mortality

**DOI:** 10.3389/fpubh.2020.00152

**Published:** 2020-04-29

**Authors:** Jian-Min Jin, Peng Bai, Wei He, Fei Wu, Xiao-Fang Liu, De-Min Han, Shi Liu, Jin-Kui Yang

**Affiliations:** ^1^Department of Respiratory and Critical Care Medicine, Beijing Tongren Hospital, Capital Medical University, Beijing, China; ^2^Department of Internal Medicine, Union Hospital, Tongji Medical College, Huazhong University of Science and Technology, Wuhan, China; ^3^Department of Critical Care Medicine, Beijing Tongren Hospital, Capital Medical University, Beijing, China; ^4^Department of Otolaryngology and Head Surgery, Beijing Tongren Hospital, Capital Medical University, Beijing, China; ^5^Department of Medicine, Beijing Tongren Hospital, Capital Medical University, Beijing, China

**Keywords:** SARS-CoV-2, COVID-19, SARS, morbidity, mortality, gender, male, female

## Abstract

**Objective:** The recent outbreak of Novel Coronavirus Disease (COVID-19) is reminiscent of the SARS outbreak in 2003. We aim to compare the severity and mortality between male and female patients with COVID-19 or SARS.

**Study Design and Setting:** We extracted the data from: (1) a case series of 43 hospitalized patients we treated, (2) a public data set of the first 37 cases of patients who died of COVID-19 and 1,019 patients who survived in China, and (3) data of 524 patients with SARS, including 139 deaths, from Beijing in early 2003.

**Results:** Older age and a high number of comorbidities were associated with higher severity and mortality in patients with both COVID-19 and SARS. Age was comparable between men and women in all data sets. In the case series, however, men's cases tended to be more serious than women's (*P* = 0.035). In the public data set, the number of men who died from COVID-19 is 2.4 times that of women (70.3 vs. 29.7%, *P* = 0.016). In SARS patients, the gender role in mortality was also observed. The percentage of males were higher in the deceased group than in the survived group (*P* = 0.015).

**Conclusion:** While men and women have the same prevalence, men with COVID-19 are more at risk for worse outcomes and death, independent of age.

## What Is New?

- This is the first preliminary study investigating the role of gender in morbidity and mortality in patients with Novel Coronavirus Disease (COVID-19).
- Men are more at risk for worse outcomes and death, independent of age, with COVID-19.
- While males and females have the same prevalence of COVID-19, male patients have a higher mortality.

## Introduction

In early December 2019, an outbreak of a novel coronavirus disease (COVID-19) occurred in Wuhan city and then rapidly spread throughout China, putting the world on alert. High-throughput sequencing has revealed a novel β-coronavirus that is currently named severe acute respiratory syndrome coronavirus 2 (SARS-CoV-2) (1), which resembles severe acute respiratory syndrome coronavirus (SARS-CoV) (2). Most patients with COVID-19 were *Mild*/*Moderate* patients who often experienced dyspnea after 1 week. *Severe* patients progressed rapidly to *Critical* conditions, which included symptoms such as acute respiratory distress syndrome (ARDS), acute respiratory failure, coagulopathy, septic shock, and metabolic acidosis.

Early identification of risk factors for *Critical* conditions is urgently needed, not only to identify the defining clinical and epidemiological characteristics with greater precision, but also to facilitate the appropriate supportive care and prompt access to the intensive care unit (ICU) if necessary.

The Chinese health authority has announced that the total number of confirmed cases on the Chinese mainland has reached 76,936, and 2,442 people have died of the disease as of Feb 23. Among the 2,442 deceased patients, most were old and two-thirds were males, though the detailed data has not been reported (3). This raises a question: Are men more susceptible to getting and dying from COVID-19?

Here, we report the clinical characteristics of a recent case series of 43 patients we treated and a public data set of the first 37 cases of those who died from COVID-19 and the 1,019 patients who survived COVID-19. We aimed to compare the severity and mortality in male and female patients with COVID-19 and to explore the most useful prognostic factor for individualized assessment. SARS-CoV-2 infection is reminiscent of the SARS-CoV outbreak in early 2003, because both viruses attack cells via the same ACE2 receptor (3). In this study, we also analyzed the data of 524 SARS patients, including 139 deaths, from Beijing in early 2003.

## Materials and Methods

### Patients and Data Sources

#### Cases Series of COVID-19

The case series analysis covers 43 patients with COVID-19 who were treated at Wuhan Union Hospital by the medical team of Beijing Tongren Hospital from January 29, 2020 to February 15, 2020.

#### Public Data Set of COVID-19

The public data set covers the first 37 cases of patients who died from COVID-19 and 1,019-cases of COVID-19 survivors from the public data set from the Chinese Public Health Science Data Center.

#### Cases Series of SARS

This study also included data of 524 SARS patients, including 139 deaths from 29 hospitals in early 2003. These patients were hospitalized in Beijing between 25 March and 22 May 2003.

Diagnosis and clinical classification criteria and treatment plan (trial version 5) of COVID-19 was launched by the National Health Committee of the People's Republic of China (http://www.nhc.gov.cn/). The clinical classification of severity is as follows: (1) *Mild*, only mild symptoms, imaging shows no pneumonia; (2) *Moderate*, with fever, respiratory tract symptoms, and imaging shows pneumonia; (3) *Severe*, meet any of the following signs: (a) respiratory distress, respiratory rate ≥ 30 beats / min; (b) in the resting state, finger oxygen saturation ≤ 93%) arterial blood oxygen partial pressure (PaO_2_/oxygen concentration (FiO_2_) ≤ 300 mmHg (1 mmHg = 0.133 kPa); (4) *Critical*, one of the following conditions: (a) respiratory failure occurs and requires mechanical ventilation, (b) Shock occurs, (c) ICU admission is required for combined organ failure.

The study protocol was approved by the Ethics Committee of Beijing Tongren Hospital, Capital Medical University.

### Statistical Analysis

Data were expressed as mean ± SD, median [interquartile range (IQR)], or percentages, as appropriate. To compare the differences between the two groups, mean values and percentages were used between the two groups by the Student *t*-test, Mann-Whitney *U*-test, or chi-square (χ^2^) test. Kaplan–Meier survival curves and the log-rank test was used for testing the survival rates between males and females. Statistical analyses were performed using the SAS software (version 9.4). *P* < 0.05 (two-tailed) was considered to be statistically significant.

## Results

### Case Series of Covid-19

**Figure 1:**
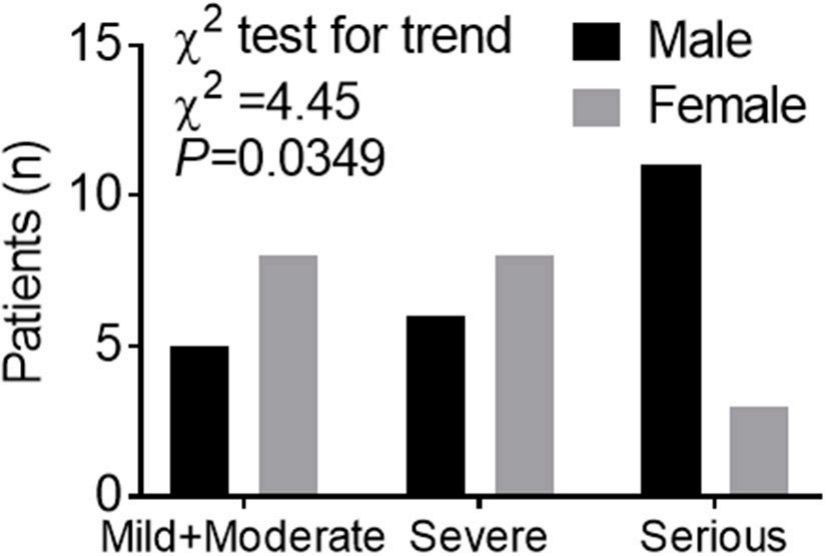
Trend data of clinical classification of severity in a *Case series of COVID-19*. Numbers of cases of men or women in different clinical classes of severity. Chi-square (χ^2^) test for trend indicated that males tend to experience more serious cases of COVID-19 than females according to the clinical classification of severity including *Mild*+*Moderate, Severe*, and *Critical*.

The demographic and clinical characteristics are shown in Table 1. The median age was 62 years (IQR, 51 to 70). Fever (95.3%) and cough (65.1%) were the most common symptoms, while diarrhea (16.3) was not common. 37.2% of patients had at least one underlying disorder (i.e., hypertension, diabetes, cardiovascular diseases, and chronic lung diseases). There is no significant difference in median age between male and female groups, but the maximum of the range of IQR is lower in male (66 years in men vs. 73 years in women). Symptoms and comorbidities were comparable between men and women. As expected, men had a higher level of hemoglobin. However, male patients also had elevated serum creatinine, white blood cells, and neutrophils. Among the 43-case series, 13 (30.2%) were diagnosed with *Mild* or *Moderate* pneumonia, while 14 (32.6%) and 16 (37.2%) were diagnosed with *Severe* and *Critical* pneumonia, respectively. Chi-square (χ^2^) test for trend indicated that men's cases of COVID-19 tended to be more serious than women's (*P* = 0.035), according to the clinical classification of severity (Figure 1).

**Table 1:** Characteristics of a Case series of COVID-19.

	**Total (*n* = 43)**	**Male (*n* = 22)**	**Female (*n* = 21)**	***P*-value**
Age, median (range) – year	62 (51–70)	59 (51–66)	63 (52–73)	0.734
**Symptoms**				
Fever – *n* (%)	41 (95.3)	21 (95.5)	20 (95.2)	0.490
Diarrhea – *n* (%)	7 (16.3)	3 (13.6)	4 (19.0)	0.946
Cough – *n* (%)	28 (65.1)	16 (72.7)	12 (57.1)	0.452
**Comorbidities –** ***n*** **(%)**				
Hypertension – *n* (%)	10 (23.3)	6 (27.3)	4 (19.0)	0.782
Diabetes history – *n* (%)	5 (11.6)	4 (18.2)	1 (0.5)	0.370
Cardiovascular diseases – *n* (%)	4 (9.3)	2 (9.1)	2 (10.0)	0.634
Chronic lung diseases – *n* (%)	1 (0.2)	1 (0.5)	0 (0)	0.981
From symptom to diagnosis, median (range) – day	12 (8–14)	12 (7–13)	12 (10–14)	0.250
Aspartate aminotransferase – IU/l	42.4 ± 18.9	43.0 ± 15.3	41.7 ± 22.6	0.872
Alanine aminotransferase – IU/l	42.8 ± 19.0	45.0 ± 18.0	40.4 ± 19.5	0.590
Alkaline phosphatase – IU/l	53.4 ± 10.6	52.6 ± 11.9	54.3 ± 9.0	0.736
Lactate dehydrogenase – IU/l	369.4 ± 132.7	414.8 ± 136.2	321.8 ± 112.9	0.064
Serum creatinine – μmol/l	**75.3** **±** **21.1**	**90.4** **±** **22.2**	**59.4** **±** **10.9**	**0.000**
Fasting Blood Glucose – mmol/l	7.3 ± 1.8	7.7 ± 2.0	6.7 ± 1.5	0.325
High sensitive C-reactive protein – mg/l	52.3 ± 27.8	58.9 ± 29.2	45.6 ± 25.3	0.323
White blood cells – ×10^9^/l	**6.8** **±** **2.2**	**7.7** **±** **2.3**	**5.8** **±** **1.5**	**0.027**
Hemoglobin – g/l	128.8 ± 13.6	139.0 ± 11.2	117.6 ± 8.6	0.000
Platelets – ×10^9^/l	225.2 ± 57.4	230.4 ± 54.1	219.6 ± 60.0	0.682
Neutrophils – ×10^9^/l	**5.4** **±** **2.2**	**6.4** **±** **2.4**	**4.3** **±** **1.3**	**0.019**
Lymphocytes – ×10^9^/l	1.0 ± 0.4	0.9 ± 0.3	1.1 ± 0.4	0.284

*Data are presented as mean ± SD, medians (interquartile ranges, IQR) and no. (%). Bold values mean statistic difference between males and females*.

### Public Data Set of COVID-19

In the deceased patients, fever (86.5%) and cough (67.6%) were common, while diarrhea was uncommon (18.9%).The median period from symptom onset to death was 13 days (ranging of IQR 11 to 18 days). Of these deceased patients, 64.9% had at least one underlying disorder (i.e., hypertension, diabetes, cardiovascular disease, or chronic obstructive pulmonary disease) (Table 2).

**Table 2:** Characteristics of a Public data set of COVID-19 and a Cases series of SARS, in 2003.

	**COVID-19**		**SARS**	
	**Deceased (*n* = 37)**	**Survived (*n* = 1019)**	**Deceased (*n* = 139)**	**Survived (*n* = 385)**
Age, median (range) – year	70 (65–81)^**^	47 (35–57)	57 (45–69)^††^	32 (24–44)
Male – *n* (%)	26 (70.3)^*^	510 (50.0)	74 (53.2)^†^	163 (42.3)
**Symptoms**				
Fever – *n* (%)	32 (86.5)		136 (97.8)	379 (98.4)
Diarrhea – *n* (%)	7 (18.9)		30 (21.6)	26 (6.8)
Cough – *n* (%)	25 (67.6)		107 (77.0)	185 (48.1)
Comorbidities – *n* (%)	24 (64.9)		79 (56.8)^††^	69 (17.9)
Hypertension – *n* (%)	18 (48.6)		64 (46.0)^††^	44 (11.4)
Diabetes history – *n* (%)	11(29.7)		30 (21.6)^††^	15 (3.9)
Cardiovascular disease – *n* (%)	8 (21.6)		40 (28.8)^††^	23 (6.0)
Chronic lung disease – *n* (%)	3 (8.1)		5 (3.6)	6 (1. 6)
From onset to death, median (range) – day	13 (11–18)		15 (10–19)	

*Data are presented as medians (interquartile ranges, IQR) and n (%)*.

* *p < 0.05*,

** *p < 0.01, vs. COVID-19 survived patients*.

† *p < 0.05*,

†† *p < 0.01, vs. SARS survived patients*.

**Figure 2:**
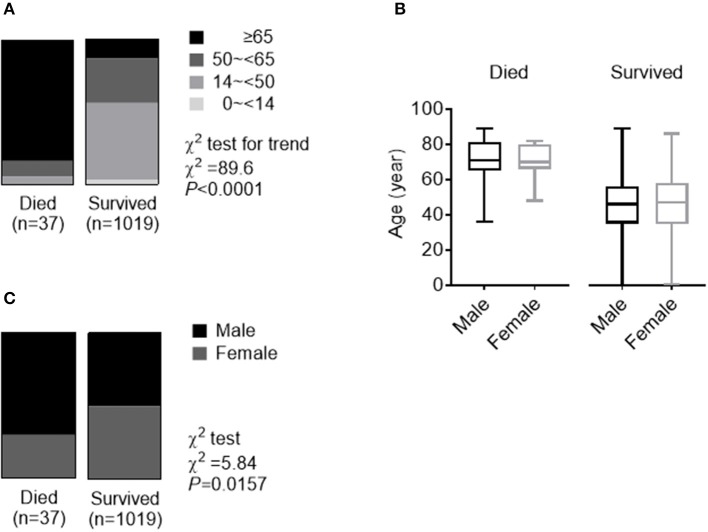
Role of age and gender in morbidity and mortality in a *Public data set of COVID-19*. **(A)** The whole spectrum of age in patients who died from and survived COVID. **(B)** Comparation of age between males and females in both patients who died from and survived COVID. **(C)** Gender distribution in both patients who died from and survived COVID.

The deceased patients were significantly older [median (IQR), 70.3 (65–81) years] and had a higher percentage of ≥65 years (83.8%), in comparison to those who survived [47 (35–57) years old and 13.2% ≥65 years]. COVID-19 was diagnosed at all ages. There were 30 (2.9%) pediatric patients (<14 years) in the group of patients who survived. None of the 37 deceased cases were pediatric patients (Table 2 and Figure 2A). Ages were comparable between men and women in both patients who deceased and survived (Figure 2B). Of the 37 deceased patients, 70.3% were men and 29.7% were woman. The number of men was 2.4 times that of women in the deceased patients. While men and women had the same susceptibility, men were more prone to dying (χ^2^ test, *P* = 0.016) (Figure 2C).

### Cases Series of SARS, in 2003

**Figure 3:**
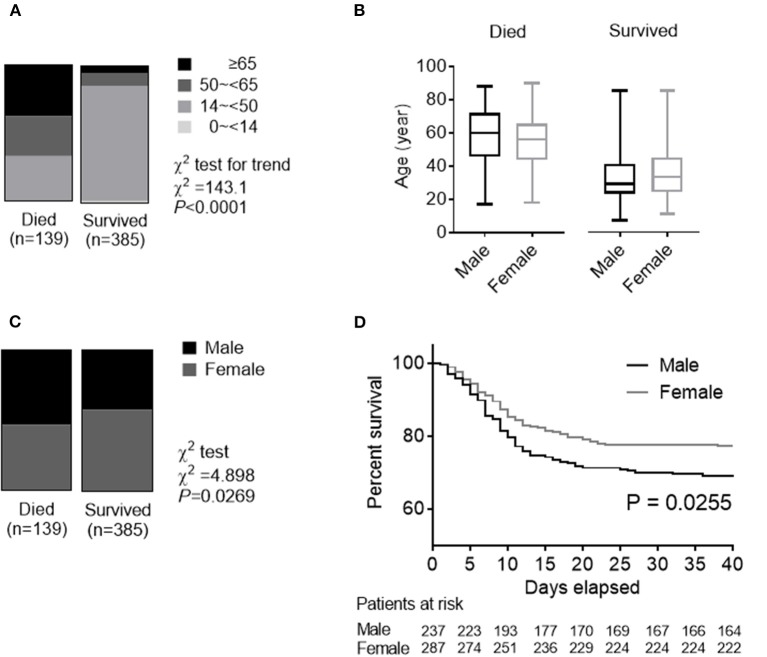
Role of age and gender in morbidity and mortality in a *Cases series of SARS, in 2003*. **(A)** The whole spectrum of age in patients who died from and survived SARS. **(B)** Comparation of age between males and females in both patients who died from and survived SARS. **(C)** Gender distribution in both patients who died from and survived SARS. **(D)** Survival analysis comparing mortality rates between male and female patients with SARS.

Between March 25 and May 22, 2003, a total of 524 SARS patients, including 139 deaths, in the Beijing area were reported from 29 hospitals enrolled in our analysis. Fever (98.4%) and cough (76.9%) were the most common symptoms, while diarrhea (6.7%) was not common. 57.0% of the patients had at least one of the concomitant diseases including hypertension, diabetes, cardiovascular diseases, and chronic lung diseases. The mean duration from self-reported symptoms to death was 15 (IQR: 10–19) days. Table 2 summarizes the clinical and biochemical characteristics of all SARS patients. The median age of the deceased patients was much higher than that of the patients who survived (57 vs. 32, *P* < 0.001). The rate of the concomitant diseases in the deceased patients was also higher than that of the patients who survived (57.0% vs. 17.9%, *P* < 0.001). While the deceased patients were significantly older than the patients who survived (Figure 3A), ages were comparable between men and women in both patients who deceased and survived with SARS (Figure 3B). The proportion of men was higher in the deceased group (53.2%) than in the group who survived (42.3%) (χ^2^ test, *P* = 0.027) (Figure 3C). Survival analysis showed that men had a significantly higher mortality rate than women (31.2 vs. 22.6%) in this hospital-based cohort (hazard ratio [95% CI] 1.47 [1.05–2.06], *P* = 0.026) (Figure 3D).

## Discussion

Coronavirus is a large family of viruses that cause illnesses ranging from the common cold to severe pneumonia, such as SARS (2) and Middle East Respiratory Syndrome (MERS) (4). SARS-CoV-2 was first identified in Wuhan, China, by the Chinese Center for Disease Control and Prevention (CDC) (1). Both epidemiological (5, 6) and clinical (7, 8) features of patients with COVID-19 have recently been reported. However, little data on the prognostic factors of COVID-19 have been reported.

In the *Case series of COVID-19*, consistent with previous reports (8–10), older patients (≥65 years old), were more likely to have a *Severe* type of COVID-19. Men tended to develop more serious cases than women, according to the clinical classification of severity. In the *Public data set of COVID-19*, we also found that the percentage of older age (≥65 years) was much higher in the deceased patients than in the patients who survived (83.8% in 37 deceased patients vs. 13.2% in 1,019 patients who survived).

The number of men is 2.4 times that of women in the deceased patients. While men and women had the same susceptibility, men were more prone to dying.

This is the first preliminary study investigating the role of gender in morbidity and mortality of SARS-CoV-2 infection. One study of 425 patients with COVID-19 indicated that 56% were males (5). Another study of 140 patients found that 50.7% were males (9). In the present study, similar susceptibility to SARS-CoV-2 between males and females was observed in 1,019 patients who survived the disease (50.0% males), collected from a public data set and in a case series of 43 hospitalized patients (51.2% males). Although the deceased patients were significantly older than the patients who survived COVID-19, ages were comparable between males and females in both the deceased and the patients who survived. Therefore, gender is a risk factor for higher severity and mortality in patients with COVID-19, independent of age and susceptibility. This gender factor, as well as higher incidences in men for most of the diseases, could correlate with a general demographic fact of a shorter life expectancy in men compared to women in China and in the world in general. Although there is no significant difference in median age between male and female groups, the maximum of the range of IQR is lower in males in the case series.

In early 2003, we participated in the Beijing Municipal Medical Taskforce of SARS (11). Here, we re-analyzed the data of a large population of 520 SARS patients, including 135 deaths in Beijing, and summarized the experience and lessons for present use, because SARS-CoV-2 and SARS-CoV attack cells via the same receptor, ACE2 (3). We have previously reported that high protein expression of ACE2 receptor in specific organs correlated with specific organ failures, indicated by corresponding clinical parameters in SARS patients (11, 12). It has been shown that circulating ACE2 levels are higher in men than in women and in patients with diabetes or cardiovascular diseases (13).

This study has some limitations. First, for severity analysis, only a case series of 43 patients with SARS-CoV-2 was included, because detailed patient information, particularly regarding clinical outcomes, was unavailable in the public data set. Second, for mortality analysis only the first 37 cases of patients who died of SARS-CoV-2 were included. Due to the urgent circumstances we are living in, there was no access to unique, homogeneous data for COVID. It could affect the analysis and any possible biased results. However, this is the first preliminary analysis investigating the role of gender in morbidity and mortality in patients with SARS-CoV-2. More clinical and basic research regarding gender and other prognostic factors for individualized assessment and treatment is needed in the future.

In conclusion, this is the first preliminary study investigating the role of gender in morbidity and mortality in patients with COVID-19. Men with COVID-19 are more at risk for worse outcomes and death, independent of age.

## Data Availability Statement

The raw data supporting the conclusions of this article will be made available by the authors, without undue reservation.

## Ethics Statement

The studies involving human participants were reviewed and approved by Ethics Committee of Beijing Tongren Hospital, Capital Medical University. Written informed consent for participation was not required for this study in accordance with the national legislation and the institutional requirements.

## Author Contributions

J-MJ, PB, WH, SL, FW, X-FL, D-MH, and J-KY collected the epidemiological and clinical data and processed statistical data. J-KY drafted the manuscript. J-MJ, SL, and J-KY revised the final manuscript. J-KY is responsible for summarizing all epidemiological and clinical data.

## Conflict of Interest

The authors declare that the research was conducted in the absence of any commercial or financial relationships that could be construed as a potential conflict of interest.

## References

[1] Zhu N Zhang D Wang W Li X Yang B Song J. A novel coronavirus from patients with Pneumonia in China, 2019. N Engl J Med. (2020) 382:727–33. 10.1056/NEJMoa2001017 31978945 PMC7092803

[2] Drosten C Gunther S Preiser W van der Werf S Brodt HR Becker S. Identification of a novel coronavirus in patients with severe acute respiratory syndrome. N Engl J Med. (2003) 348:1967–76. 10.1056/NEJMoa030747 12690091

[3] National Health Commission of PRC Daily Briefing on Novel Coronavirus Cases in China. (2020). Available online at: http://ennhcgovcn/2020-02/23/c_76779htm

[4] Zaki AM van Boheemen S Bestebroer TM Osterhaus AD Fouchier RA. Isolation of a novel coronavirus from a man with pneumonia in Saudi Arabia. N Engl J Med. (2012) 367:1814–20. 10.1056/NEJMoa1211721 23075143

[5] Li Q Guan X Wu P Wang X Zhou L Tong Y. Early Transmission dynamics in Wuhan, China, of novel coronavirus-infected pneumonia. N Engl J Med. (2020) 382:1199–207. 10.1056/NEJMoa2001316 31995857 PMC7121484

[6] Chan JF Yuan S Kok KH To KK Chu H Yang J. A familial cluster of pneumonia associated with the 2019 novel coronavirus indicating person-to-person transmission: a study of a family cluster. Lancet. (2020) 395:514–23. 10.1016/S0140-6736(20)30154-9 31986261 PMC7159286

[7] Huang C Wang Y Li X Ren L Zhao J Hu Y. Clinical features of patients infected with 2019 novel coronavirus in Wuhan, China. Lancet. (2020) 395:497–506. 10.1016/S0140-6736(20)30183-5 31986264 PMC7159299

[8] Chen N Zhou M Dong X Qu J Gong F Han Y. Epidemiological and clinical characteristics of 99 cases of 2019 novel coronavirus pneumonia in Wuhan, China: a descriptive study. Lancet. (2020) 395:507–13. 10.1016/S0140-6736(20)30211-7 32007143 PMC7135076

[9] Zhang JJ Dong X Cao YY Yuan YD Yang YB Yan YQ. Clinical characteristics of 140 patients infected by SARS-CoV-2 in Wuhan, China. Allergy. (2020). 10.1111/all.14238. [Epub ahead of print]. 32077115

[10] Wang D Hu B Hu C Zhu F Liu X Zhang J. Clinical characteristics of 138 hospitalized patients with 2019 novel coronavirus-infected pneumonia in Wuhan, China. JAMA. (2020). 10.1001/jama.2020.1585. [Epub ahead of print]. 32031570 PMC7042881

[11] Yang JK Feng Y Yuan MY Yuan SY Fu HJ Wu BY. Plasma glucose levels and diabetes are independent predictors for mortality and morbidity in patients with SARS. Diabet Med. (2006) 23:623–8. 10.1111/j.1464-5491.2006.01861.x 16759303

[12] Yang JK Lin SS Ji XJ Guo LM. Binding of SARS coronavirus to its receptor damages islets and causes acute diabetes. Acta Diabetol. (2010) 47:193–9. 10.1007/s00592-009-0109-4 19333547 PMC7088164

[13] Patel SK Velkoska E Burrell LM. Emerging markers in cardiovascular disease: where does angiotensin-converting enzyme 2 fit in? Clin Exp Pharmacol Physiol. (2013) 40:551–9. 10.1111/1440-1681.12069 23432153

[14] Jin JM Bai P He W Wu F Liu XF Han DM Gender differences in patients with COVID-19: focus on severity and mortality. medRxiv [Preprint]. (2020). 10.1101/2020.02.23.20026864 PMC7201103 32411652

